# Mazzini’s Views of Death

**Published:** 1892-01

**Authors:** 


### MAZZINI'S VIEWS OF DEATH.

In *The Century* magazine for November, occur these expressions of Mazzini, the Italian patriot, in some letters written to members of an English family with whom he was very intimate, in regard to death and a future life, the death of a member of the family being the occasion; " Remember, for God's sake, that there is no such thing as death for all that is best in us; that what people call death is only a transformation and step onward in life. Love is a voucher for immortality. We would not scatter a single flower on a tomb if there was not an instinct in the soul teaching us that our love pleases the cherished one who is buried beneath, and depend upon me there is more truth discovered by these flashes of the virgin soul than by all the dim, painfully elaborated lanterns of analysis and reasoning knowledge."

And again: " Let you all feel, as I shall, her presence more than ever. Let you all believe—as you believe in my undying affection—that death is the cradle of a new, purer and happier life. It is so. God knows I would not give at such a moment a mere poetical instinct as a consolation. I know it is so. Every departure of loved beings has made me feel so more and more. Your mother is living, loving, wanting love; longing for your rising (sometime) calmly and trustfully to her, and rewarded for the love she had, for the truth she did and wished to do, with some more power to help you on, to influence you with holy, virtuous thoughts."

